# Impact of Intermittent Hypoxia Related to Obstructive Sleep Apnoea Syndrome on Low-Grade Inflammation in Hypertensive Patients: Potential Implications for Cardiovascular Risk

**DOI:** 10.3390/life14050592

**Published:** 2024-05-06

**Authors:** Matthieu Hein, Benjamin Wacquier, Matteo Conenna, Jean-Pol Lanquart, Camille Point

**Affiliations:** 1 Hôpital Universitaire de Bruxelles, Service de Psychiatrie et Laboratoire du Sommeil, Université Libre de Bruxelles, ULB, 1070 Brussels, Belgium; benjamin.wacquier@hubruxelles.be (B.W.); matteo.conenna@hubruxelles.be (M.C.); secmed.psy.erasme@hubruxelles.be (J.-P.L.); camille.point@hubruxelles.be (C.P.); 2 Laboratoire de Psychologie Médicale et Addictologie (ULB312), Université Libre de Bruxelles, ULB, 1020 Brussels, Belgium

**Keywords:** C-reactive protein, intermittent oxygen desaturation, sleep-related breathing disorder, high blood pressure

## Abstract

There is evidence for a particular relationship between low-grade inflammation (LGI) and intermittent hypoxia (IH) related to obstructive sleep apnoea syndrome (OSAS). However, despite the potential deleterious cardiovascular consequences associated with this LGI in hypertensive patients, few studies have investigated the impact of IH related to OSAS on CRP levels in this subpopulation. In total, 1404 hypertensive patients were selected retrospectively from the Sleep Laboratory database. CRP levels ≥3 mg/L but <10 mg/L were used as cut-offs to identify hypertensive patients with LGI. Logistic regressions were conducted to examine the risk of LGI associated with IH related to OSAS in hypertensive patients. LGI was frequent (33.8%) in hypertensive patients. After adjustment for confounders, multivariate logistic regressions revealed that only moderate to severe OSAS (apnoea–hypopnoea index ≥ 15/h) with high IH (oxygen desaturation index ≥ 15/h) [OR 1.51 (95% CI 1.06–2.14)] was significantly associated with LGI in hypertensive patients (*p*-value = 0.045). Consistent with our hypothesis, our results demonstrated the existence of a particular subtype of hypertensive patients at high cardiovascular risk characterised by the presence of LGI induced by IH hypoxia related to moderate to severe OSAS, which justifies the establishment of adequate management of this pathology to allow better cardiovascular prevention in this subpopulation.

## 1. Introduction

In the literature, there are many arguments in favour of a particular relationship between hypertension and some inflammatory markers such as C-reactive protein (CRP) [1]. Indeed, hypertensive patients have higher CRP levels than normotensive subjects [2]. In addition, the presence of high CRP levels is associated with a higher risk of developing hypertension in the general population, whereas individuals with high CRP levels have greater hypertension-related complications than those with low CRP levels [3,4]. However, in individuals with high CRP levels, the occurrence of this higher risk of developing hypertension and having hypertension-related complications may be explained by the activation of some deleterious pathophysiological mechanisms: alteration of the renin–angiotensin–aldosterone system, plaque remodelling, oxidative stress, endothelial dysfunction, opsonisation of oxidized LDL cholesterol, prothrombotic state, activation of complement cascade and vascular wall damage [5]. Moreover, in hypertensive patients, the presence of high CRP levels appears to be associated with a more frequent development of comorbid cardiovascular diseases and a less favourable cardiovascular outcome [6,7,8] through the occurrence or worsening of some specific complications related to the pathophysiology of hypertension that are deleterious to the cardiovascular system (vascular stiffness, atherosclerosis and end-organ damage) [9]. In addition, it has been shown that the reduction in these high CRP levels following the use of statins and/or some antihypertensive medications may have a beneficial effect in terms of cardiovascular prevention in hypertensive patients [10,11]. Thus, to avoid these deleterious cardiovascular consequences related to high CRP levels (defined in the literature as ≥3 mg/L but <10 mg/L) [12], it seems necessary to identify the conditions associated with the presence of low-grade inflammation in hypertensive patients in order to allow the establishment of better cardiovascular prevention strategies in this particular subpopulation.

In the general population, it has been shown that some sleep disorders are associated with the presence of high CRP levels [13,14,15]. Among these sleep disorders, obstructive sleep apnoea syndrome (OSAS) seems to play a central role in the development of this chronic low-grade inflammation [16,17]. Indeed, sleep alterations specific to OSAS (intermittent hypoxia, excessive sleep fragmentation and sleep deprivation) may promote inadequate activation of the inflammatory cascade inducing the occurrence of high CRP levels [16,17]. However, even if this particular relationship between OSAS and high CRP levels has been demonstrated in the general population [18], there are few data on this specific problem in hypertensive patients despite the frequent co-occurrence between these two pathologies [19,20]. Furthermore, the only studies currently available mainly investigated the impact of OSAS severity (based only on the apnoea–hypopnoea index [AHI]) on CRP levels in hypertensive patients [21,22,23]. However, in the literature, some studies seem to indicate that in the case of OSAS, the polysomnographic parameters related to intermittent hypoxia (such as the oxygen desaturation index [ODI]) could be more specific predictors than the AHI for the presence of high CRP levels [24,25]. Furthermore, alongside this potential central role played in the development of low-grade inflammation, these polysomnographic parameters related to intermittent hypoxia also appear to be directly involved in the activation of other mechanisms harmful to the cardiovascular system (sympathetic hyperexcitation, production of reactive oxygen species, metabolic dysregulation and endothelial dysfunction) [26]. These elements therefore seem to indicate that rather than focusing solely on the AHI, it would also be interesting to study the impact of intermittent hypoxia related to OSAS on CRP levels in hypertensive patients in order to enable a better understanding of less favourable cardiovascular outcomes associated with low-grade inflammation in this particular subpopulation.

This study aimed to investigate the impact of intermittent hypoxia related to OSAS on CRP levels in hypertensive patients. The hypothesis of this study was that unlike other conditions (mild OSAS or moderate to severe OSAS with low intermittent hypoxia), only moderate to severe OSAS with high intermittent hypoxia is significantly associated with low-grade inflammation (CRP levels ≥3 mg/L but <10 mg/L) in hypertensive patients. The goal of this approach was to highlight a particular subtype of hypertensive patients at high cardiovascular risk characterised by the presence of low-grade inflammation induced by high intermittent hypoxia related to moderate to severe OSAS to open new management perspectives for this particular subpopulation.

## 2. Materials and Methods

### 2.1. Population

**Figure 1:**
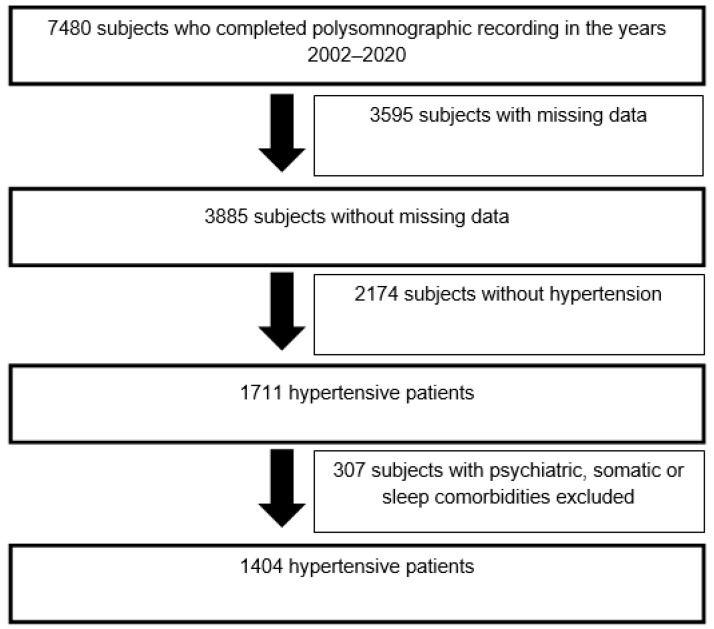
Selection diagram of hypertensive patients included in this study.

From the database of polysomnographic recordings performed between 1 January 2002 and 31 December 2020 at the Erasme Hospital Sleep Laboratory, 1404 hypertensive patients according to the diagnostic criteria of the World Health Organization were selected retrospectively based on the inclusion and exclusion criteria defined for this study (Table 1) [27,28]. These hypertensive patients had stayed consecutively at the Erasme Hospital Sleep Laboratory to benefit from a complete assessment of their sleep (Figure 1). Only hypertensive patients were recruited in this study because the aim was to focus on this particular subpopulation where cardiovascular outcome may be negatively impacted by the presence of high CRP levels [6,7,8]. The Supplementary Data—Annex S1 contains a detailed description of the recruitment procedure for these hypertensive patients from outpatient sleep medicine consultations to admission for polysomnographic recordings at the Sleep Laboratory.

### 2.2. Methods

#### 2.2.1. Medical and Psychiatric Assessment

Upon admission to the Sleep Laboratory, all hypertensive patients selected for this study benefited from a complete somatic check-up (clinical interview, review of medical records and complementary tests [blood test, urine analysis, electrocardiogram and electroencephalogram]) in order to screen for their potential medical comorbidities. In addition, during this somatic assessment, repeated measurements of blood pressure and a check of potential antihypertensive medications prescribed were carried out to confirm the diagnosis of hypertension and its status (untreated, controlled and uncontrolled) in all patients selected for this study (Supplementary Data—Annex S2). CRP levels used in this study were defined using blood tests performed during this somatic check-up (Roche CRP4_Cobas with minimum detection level of 0.3 mg/L) [29] in order to exclude patients with CRP levels ≥10 mg/L that may be induced by other pathological conditions (such as infections or inflammatory diseases) [30]. Based on these measured CRP levels, low-grade inflammation was defined as absent when CRP levels were <3 mg/L and as present when CRP levels were ≥3 mg/L but <10 mg/L [31].

Afterwards, to allow identification of their potential psychiatric comorbidities, all hypertensive patients selected for this study benefited from a systematic psychiatric interview based on diagnostic criteria from DSM-IV-TR (before 2013) and DSM 5 (after 2013) by a psychiatrist assigned to the Sleep Laboratory [32,33].

Finally, the subjective complaints of all hypertensive patients selected for this study were assessed using a series of self-questionnaires: Beck Depression Inventory (reduced to 13 items), Epworth Sleepiness Scale and Insomnia Severity Index (Supplementary Data—Annex S3) [34,35,36].

#### 2.2.2. Sleep Evaluation and Study

In all hypertensive patients selected for this study, sleep habits and sleep-related complaints were systematically investigated by a psychiatrist assigned to the Sleep Laboratory during a semi-structured interview to identify potential symptoms suggestive of sleep pathologies. After this specific interview focused on sleep, all these hypertensive patients underwent polysomnographic recording meeting the criteria of the American Academy of Sleep Medicine [37]. The Supplementary Data—Annex S4 contains a detailed description of the stay conditions at the Sleep Laboratory and polysomnography montage used for the recordings. Finally, based on recommendations from the American Academy of Sleep Medicine (Supplementary Data—Annex S5), technical reports for these polysomnographic recordings were produced after visual scoring by specialised technologists [38,39].

Concerning obstructive respiratory events, obstructive apnoeas were scored if the decrease in air flow was ≥90% for at least 10 s, whereas obstructive hypopnoeas were scored if the decrease in airflow was ≥30% for at least 10 seconds with a decrease in oxygen saturation of 3% or followed by micro-arousal [40]. The AHI corresponds to the total number of obstructive apnoeas and hypopnoeas divided by the period of sleep in hours [40]. Concerning the parameters related to intermittent hypoxia, oxygen desaturations were scored if oxygen saturation showed a ≥3% decrease from baseline [40]. The ODI corresponds to the total number of oxygen desaturations divided by the period of sleep in hours [40].

Following this comprehensive sleep assessment, all hypertensive patients selected for this study therefore benefited from a systematic diagnosis of their potential comorbid sleep pathologies: insomnia disorder, OSAS (absent [AHI < 5/h], mild [AHI 5–14/h], moderate to severe [AHI ≥15/h]), intermittent hypoxia (low [ODI < 15/h], high [ODI ≥ 15/h]), moderate to severe periodic limb movement syndrome (periodic limb movement index ≥ 15/h), restless legs syndrome and short sleep duration (<6 h) [41,42,43,44,45,46].

### 2.3. Statistical Analyses

Statistical analyses were performed using Stata 14. The normal distribution of the data was verified using histograms, boxplots and quantile–quantile plots. The equality of variances was checked using Levene’s test. 

During analyses, hypertensive patients without low-grade inflammation (CRP levels < 3 mg/L) were included in the control group and hypertensive patients with low-grade inflammation (CRP levels ≥ 3 mg/L but <10 mg/L) were included in the subject group [12,31].

Continuous data were described by their median (P25–P75) and analysed by Wilcoxon tests (non-parametric tests) since most of these data were not distributed symmetrically. Concerning categorical data, percentages were used for descriptive analyses and Chi² tests for group comparisons.

Univariate logistic regression analyses were used to investigate the risk of low-grade inflammation associated with OSAS status (categorised: no, mild, moderate to severe with low intermittent hypoxia, moderate to severe with high intermittent hypoxia) and potential confounding factors (Supplementary Data—Annex S6). Afterwards, during multivariate logistic regression analyses, risk of low-grade inflammation associated with OSAS status was adjusted only for significant confounding factors during univariate analyses. In order to take into account the potential impact of these confounding factors, risk of low-grade inflammation associated with OSAS status was therefore adjusted after hierarchical introduction:

- For gender, the presence of obesity and smoking status in model 1.
- For gender, the presence of obesity, smoking status, presence of type 2 diabetes, presence of dyslipidaemia and aspirin therapy in model 2.
- For gender, the presence of obesity, smoking status, presence of type 2 diabetes, presence of dyslipidaemia, aspirin therapy and Epworth Sleepiness Scale scores in model 3.

Finally, after this hierarchical introduction of confounding factors significantly associated with low-grade inflammation, the adequacy of the final multivariate model was verified by the Hosmer and Lemeshow test and the specificity of the model by Link Test. 

Based on the conditions of use of the multivariate logistic regression analyses characterized by the need to have at least 10 subjects per predictor included in models [47,48], each of the two groups of hypertensive patients for this study had to contain at least 170 subjects (10 subjects * 17 potential predictors) to ensure the validity of the analyses performed, which was largely achieved in this study.

A *p*-value < 0.05 was considered significant.

### 2.4. Data Collected

The data collected for this study were sleep latency, sleep efficiency, sleep period time, total sleep time, % stage 1, %stage 2, % stage 3, % REM, % wake after sleep onset, REM latency, number of awakenings, micro-arousal index, apnoea–hypopnoea index, oxygen desaturation index, total time under 90% oxygen, index of periodic limb movements, sex, age, body mass index, systolic blood pressure, diastolic blood pressure, CRP levels, total cholesterol, triglyceride levels, HDL cholesterol levels, blood glucose at admission, smoking, alcohol consumption, caffeine consumption, antihypertensive treatments, antidiabetic treatments, cholesterol-lowering treatments, aspirin therapy, psychiatric history, current psychiatric pathologies, somatic history, current somatic pathologies, history of sleep pathologies, current sleep pathologies, Beck Depression Inventory score (13 items), Epworth Sleepiness Scale score and Insomnia Severity Index score. Only patients without missing data for these different variables were included in this study.

## 3. Results

The summary of the main results of this study is available in Table 2.

### 3.1. Polysomnographic Data (Table 3)

Compared to those without low-grade inflammation, hypertensive patients with low-grade inflammation present a decrease in the index of periodic limb movements during sleep (2 [0–12] vs. 1 [0–10], *p* = 0.033) and an increase in REM latency (84.2 [60.0–132.0] vs. 87.5 [60.9–153.8)], *p* = 0.036), ODI (3 [1–10] vs. 4 [1–15], *p* = 0.001) and total time under 90% of oxygen saturation (4.3 [0.0–36.5] vs. 13.0 [0.5–78.0], *p* < 0.001). There were no significant differences for other polysomnographic parameters.

**Table 3:** Polysomnographic data (n = 1404).

	Whole Sample (n = 1404)	Subjects without LGI (n = 929)	Subjects with LGI (n = 475)	*p*-Value
Sleep latency (min)	26.3 (14.4–50.5)	25.0 (14.3–49.0)	28.0 (14.7–51.0)	0.256
Sleep efficiency (%)	76.6 (66.2–83.9)	76.8 (66.7–83.9)	76.3 (65.2–84.0)	0.680
Sleep period time (min)	449.5 (408.5–482.8)	451.0 (410.5–485.5)	445.7 (406.0–478.5)	0.173
Total sleep time (min)	376.0 (326.4–416.5)	379.0 (327.5–418.0)	373.0 (322.0–414.5)	0.231
% stage 1	8.1 (5.4–11.5)	8.2 (5.6–11.2)	7.9 (5.2–11.8)	0.470
% stage 2	54.9 (47.2–61.4)	55.0 (47.4–61.3)	54.8 (46.2–61.7)	0.755
% Stage 3	2.3 (0.1–7.9)	2.3 (0.1–7.6)	2.6 (0.2–8.2)	0.325
% REM	15.5 (10.9–19.3)	15.6 (11.3–19.4)	15.2 (10.2–19.3)	0.115
REM latency (min)	85.0 (60.0–137.5)	84.2 (60.0–132.0)	87.5 (60.9–153.8)	0.036
% wake after sleep onset	14.0 (8.4–22.6)	13.9 (8.4–22.4)	14.2 (8.1–23.2)	0.987
Number of awakenings	32 (22–47)	32 (22–47)	32 (21–48)	0.487
Micro-arousal index	13 (8–22)	13 (8–21)	14 (8–25)	0.119
Apnoea–hypopnoea index	7 (2–22)	7 (2–21)	8 (2–28)	0.151
Oxygen desaturation index	3 (1–11)	3 (1–10)	4 (1–15)	0.001
Total time under 90% of SaO_2_ (min)	6.3 (0.3–49.8)	4.3 (0.0–36.5)	13.0 (0.5–78.0)	<0.001
PLMS index	2 (0–11)	2 (0–12)	1 (0–10)	0.033
	Median (P25–P75)	Median (P25–P75)	Median (P25–P75)	Wilcoxon test

LGI = low-grade inflammation, REM = rapid eye movement sleep, SaO_2_ = oxygen saturation, PLMS = periodic limb movements during sleep.

### 3.2. Univariate Analyses (Table 4)

In our sample, low-grade inflammation was present in 33.8% of hypertensive patients (n = 475). Female gender (*p* < 0.001), obesity (*p* < 0.001), smoking (*p* = 0.018), type 2 diabetes (*p* < 0.001), dyslipidaemia status (*p* = 0.001), aspirin therapy (*p* = 0.021), Epworth Sleepiness Scale scores (*p* = 0.036) and OSAS status (*p* = 0.03) were significantly associated with a higher risk of low-grade inflammation in hypertensive patients. Moreover, hypertensive patients with low-grade inflammation had higher body mass indexes (28.4 [25.3–32.1] vs. 32.2 [28.4–37.1], *p* < 0.001)—CRP levels (1.2 [0.1–1.9] vs. 4.9 [3.8–7.0], *p* < 0.001)—Epworth Sleepiness Scale scores (9 [5–13] vs. 9 [6–14], *p* = 0.005) than those without low-grade inflammation. There were no significant differences for other demographic parameters.

**Table 4:** Univariate analyses (n = 1404).

Variables	Categories	%	Subjects without LGI	Subjects with LGI	*p*-Value Chi²	OR (CI 95%)	*p*-Value
Gender	Female (n = 435) male (n = 969)	31.0% 69.0%	25.2% 74.8%	42.3% 57.7%	<0.001	1 0.46 (0.36 to 0.58)	<0.001
Age (years)	<50 (n = 574) ≥50 (n = 830)	40.9% 59.1%	40.8% 59.2%	41.1% 58.9%	0.926	1 0.99 (0.79 to 1.24)	0.926
BMI (kg/m²)	<30 (n = 738) ≥30 (n = 666)	52.5% 47.5%	62.1% 37.9%	33.9% 66.1%	<0.001	1 3.20 (2.54 to 4.03)	<0.001
Smoking	No (n = 1131) Yes (n = 273)	80.6% 19.4%	82.4% 17.6%	77.1% 22.9%	0.018	1 1.39 (1.06 to 1.82)	0.018
Alcohol	No (n = 887) Yes (n = 517)	63.2% 36.8%	62.3% 37.7%	64.8% 35.2%	0.355	1 0.90 (0.71 to 1.13)	0.355
Caffeine	No (n = 279) Yes (n = 1125)	19.9% 80.1%	21.1% 78.9%	17.5% 82.5%	0.107	1 1.26 (0.95 to 1.68)	0.108
Type 2 diabetes	No (n = 1106) Yes (n = 298)	78.8% 21.2%	81.6% 18.4%	73.3% 26.7%	<0.001	1 1.62 (1.24 to 2.10)	<0.001
Dyslipidaemia status	No (n = 526) Untreated (n = 488) Treated (n = 390)	37.5% 34.8% 27.7%	37.6% 32.0% 30.4%	37.3% 40.2% 22.5%	0.001	1 1.27 (0.98 to 1.64) 0.75 (0.56 to 0.99)	0.001
Hypertension status	Untreated (n = 533) Controlled (n = 527) Uncontrolled (n = 344)	38.0% 37.5% 24.5%	39.3% 37.9% 22.8%	35.4% 36.8% 27.8%	0.104	1 1.08 (0.84 to 1.40) 1.35 (1.02 to 1.80)	0.104
Number of antihypertensive treatments	0 (n = 533) 1 (n = 510) 2 (n = 236) ≥3 (n = 125)	38.0% 36.3% 16.8% 8.9%	39.3% 36.1% 16.6% 8.0%	35.4% 36.8% 17.3% 10.5%	0.316	1 1.13 (0.88 to 1.47) 1.16 (0.84 to 1.60) 1.45 (0.97 to 2.16)	0.318
Cardiovascular comorbidities	No (n = 1119) Yes (n = 285)	79.7% 20.3%	79.9% 20.1%	79.4% 20.6%	0.825	1 1.03 (0.78 to 1.36)	0.825
Aspirin therapy	No (n = 1084) Yes (n = 320)	77.2% 22.8%	75.4% 34.6%	80.8% 19.2%	0.020	1 0.72 (0.55 to 0.95)	0.021
OSAS status	No (n = 566) Mild (n = 362) Moderate to severe with ODI < 15/h (n = 217) Moderate to severe without ODI ≥ 15/h (n = 259)	40.3% 25.8% 15.5% 18.4%	40.6% 27.3% 16.3% 15.8%	39.8% 22.7% 13.9% 23.6%	0.003	1 0.85 (0.64 to 1.13) 0.87 (0.62 to 1.22) 1.52 (1.12 to 2.05)	0.003
Insomnia disorder	No (n = 469) Sleep deprivation alone (n = 314) Insomnia without short sleep duration (n = 367) Insomnia with short sleep duration (n = 254)	33.4% 22.4% 26.1% 18.1%	34.8% 21.9% 25.7% 17.6%	30.7% 23.4% 27.0% 18.9%	0.508	1 1.21 (0.89 to 1.64) 1.18 (0.89 to 1.58) 1.21 (0.88 to 1.68)	0.509
Sleep movement disorders	No (n = 1112) Moderate to severe PLMs alone (n = 110) RLS alone or combined with PLMs (n = 182)	79.2% 7.8% 13.0%	78.2% 8.0% 13.8%	81.3% 7.6% 11.1%	0.325	1 0.91 (0.60 to 1.39) 0.77 (0.55 to 1.09)	0.326
ESS	<11 (n = 843) ≥11 and <14 (n = 245) ≥14 (n = 316)	60.0% 17.5% 22.5%	61.8% 17.8% 20.4%	56.6% 16.8% 26.6%	0.035	1 1.03 (0.76 to 1.40) 1.42 (1.08 to 1.85)	0.036
Major depression	No (n = 783) Remitted (n = 295) Current (n = 326)	55.8% 21.0% 23.2%	56.8% 20.8% 22.4%	53.7% 21.5% 24.8%	0.485	1 1.09 (0.82 to 1.45) 1.17 (0.90 to 1.54)	0.485
LGI	No (n = 929) Yes (n = 475)	66.2% 33.8%					
	Median (P25–P75)				Wilcoxon test		
Age (years)	52 (45–60)		53 (45–60)	52 (45–59)	0.380		
BMI (kg/m²)	29.6 (26.2–33.8)		28.4 (25.3–32.1)	32.2 (28.4–37.1)	<0.001		
CRP (mg/L)	1.9 (1.0–3.8)		1.2 (0.1–1.9)	4.9 (3.8–7.0)	<0.001		
ESS	9 (6–13)		9 (5–13)	9 (6–14)	0.005		
ISI	14 (9–18)		14 (9–17)	14 (10–18)	0.164		
BDI	4 (2–8)		4 (2–8)	4 (2–8)	0.056		

LGI = low-grade inflammation, BMI = body mass index, OSAS = obstructive sleep apnoea syndrome, ODI = oxygen desaturation index, PLMs = periodic limb movements during sleep, RLS = restless legs syndrome, CRP = C-reactive protein, ESS = Epworth Sleepiness Scale, ISI = Insomnia Severity Index, BDI = Beck Depression Inventory.

### 3.3. Multivariate Analyses (Table 5)

After adjustment by hierarchical introduction of significant confounding factors identified during the univariate analyses, multivariate logistic regression analyses demonstrated that unlike mild OSAS [OR 0.95 (95% CI 0.70–1.30)] and moderate to severe OSAS with low intermittent hypoxia [OR 0.93 (95% CI 0.64–1.35)], only moderate to severe OSAS with high intermittent hypoxia [OR 1.51 (95% CI 1.06–2.14)] was significantly associated with a higher risk of low-grade inflammation in hypertensive patients (*p*-value = 0.045).

**Table 5:** Multivariate analyses (n = 1404).

Variables	Model 1 OR Adjusted (CI 95%)	*p*-Value	Model 2 OR Adjusted (CI 95%)	*p*-Value	Model 3 OR Adjusted (CI 95%)	*p*-Value
OSAS No Mild Moderate to severe with ODI < 15/h Moderate to severe without ODI ≥ 15/h	1 0.92 (0.68 to 1.25) 0.88 (0.61 to 1.27) 1.50 (1.06 to 2.13)	0.023	1 0.95 (0.70 to 1.30) 0.93 (0.64 to 1.34) 1.51 (1.06 to 2.14)	0.041	1 0.95 (0.70 to 1.30) 0.93 (0.64 to 1.35) 1.51 (1.06 to 2.14)	0.045

Model 1 = model adjusted for gender, BMI and smoking. Model 2 = model adjusted for gender, BMI, smoking, type 2 diabetes, dyslipidaemia and aspirin therapy. Model 3 = model adjusted for gender, BMI, smoking, type 2 diabetes, dyslipidaemia, aspirin therapy and ESS scores. BMI = body mass index, OSAS = obstructive sleep apnoea syndrome, ODI = oxygen desaturation index.

## 4. Discussion

In our study, we highlighted that in hypertensive patients, the prevalence of low-grade inflammation (CRP levels ≥3 mg/L but <10 mg/L) was 33.8%, which seems to indicate that this problem is more important in this particular subpopulation than in the general population (17.6–21.6%) [49,50]. However, in hypertensive patients, the presence of some specific pathophysiological mechanisms favouring inadequate activation of the inflammatory cascade (activation of complement, change in the phenotype of circulating immune cells and activation of inflammasome) could provide a better understanding of this difference in the prevalence of low-grade inflammation with the general population [51]. Moreover, this prevalence is higher than that of 21.1% highlighted by Iwashima et al. (2007) [52] and lower than that of 50.2% demonstrated by Cortez et al. (2016) [6], which could be explained by the application of different inclusion and exclusion criteria in these two studies. First, in the study of Iwashima et al. (2007) [52], individuals with secondary hypertension were excluded. However, the application of this exclusion criteria may have led to an underestimation of the prevalence of low-grade inflammation since some of the most frequent causes of secondary hypertension (such as OSAS) are associated with alterations in inflammatory mechanisms characterized by the occurrence of high CRP levels [21,53]. Second, in the study of Cortez et al. (2016) [6], the main inclusion criterion was the presence of resistance to antihypertensive medications. However, the application of this inclusion criterion may have favoured an overestimation of the prevalence of low-grade inflammation since some of the pathophysiological alterations related to high CRP levels (alteration of the renin–angiotensin–aldosterone system, oxidative stress and endothelial dysfunction) seem to play a major role in the development of resistance to antihypertensive medications [5,54,55]. Furthermore, consistent with the limited data available in the literature, the prevalence of low-grade inflammation demonstrated in our study is similar to that highlighted by Rizzo et al. (2009) (34.7%) and Zdrojewski et al. (2006) (32.0%) [56,57], which seems to confirm that low-grade inflammation is present in about a third of hypertensive patients. However, in this particular subpopulation, the presence of low-grade inflammation is associated with less favourable cardiovascular prognosis, which justifies the implementation of adequate cardiovascular prevention strategies to avoid these deleterious cardiovascular consequences related to high CRP levels in hypertensive patients [58].

Furthermore, we demonstrated that the prevalence of OSAS was 59.7% in our sample of hypertensive patients, which seems to confirm the frequent occurrence of this pathology in this particular subpopulation [59]. Yet, consistent with the literature, we highlighted that mild OSAS was not associated with the presence of a low-grade inflammation level in hypertensive patients. Indeed, in this particular subpopulation, there are arguments in favour of a potential implication of OSAS severity in the occurrence of low-grade inflammation since only moderate to severe OSAS seems to be associated with the presence of high CRP levels in hypertensive patients [20,21]. However, similarly to some subpopulations [60,61,62], this assessment of OSAS severity based solely on the AHI does not appear to be a sufficiently specific predictor for the presence of low-grade inflammation in hypertensive patients. Indeed, in our study, we demonstrated that, unlike moderate to severe OSAS with high intermittent hypoxia, moderate to severe OSAS with low intermittent hypoxia is not associated with low-grade inflammation in hypertensive patients, which seems to confirm that rather than focusing solely on the AHI, it is also necessary to take into account the polysomnographic markers related to intermittent hypoxia (such as the ODI) when assessing the impact of OSAS severity on CRP levels [60,61,62]. In addition, in hypertensive patients with moderate to severe OSAS, this central role played by intermittent hypoxia in the occurrence of low-grade inflammation could be explained by the fact that unlike hypopnoeas associated with micro-arousals, only hypopnoeas associated with oxygen desaturation appear to be involved in the development of cardiovascular risk factors [63]. Pathophysiologically, this occurrence of low-grade inflammation associated only with obstructive respiratory events inducing intermittent hypoxia may explain the activation of several deleterious mechanisms (induction of oxidative stress, reactive oxygen species production, activation of critical proinflammatory transcription factors, elevated expression of proinflammatory cytokines and chemokines, recruitment and infiltration of proinflammatory M1 macrophages) favouring inadequate activation of the inflammatory cascade [16,17,64]. Thus, in this study, we demonstrated the existence of a particular subtype of hypertensive patients at high cardiovascular risk characterised by the presence of low-grade inflammation induced by high intermittent hypoxia related to moderate to severe OSAS, which justifies systematic screening for this pathology in hypertensive patients with low-grade inflammation [65].

Among the treatments currently available, continuous positive airway pressure (CPAP) involves air that is pressurised by an electronic device and delivered during sleep via a nasal or orinasal mask, acting as a pneumatic stent of the airway that suppresses the hypoxic episodes related to OSAS [66]. Moreover, in the literature, it has been shown that in hypertensive patients with moderate to severe OSAS, the adequate use of CPAP (≥4 h per night) could allow a better cardiovascular outcome through an indirect improvement in atherosclerosis following the inhibition of inflammatory processes (such as high CRP levels) related to OSAS [67,68]. Furthermore, alongside this adequate use of CPAP (≥4 h per night), the duration of CPAP treatment could also play a major role in reducing cardiovascular risk for hypertensive patients with moderate to severe OSAS since, according to available studies, CRP levels appear to show a significant reduction after only a minimum of 3 months of CPAP treatment [69,70]. Moreover, complementary to this CPAP treatment in hypertensive patients with moderate to severe OSAS, the establishment of both lifestyle changes (such as weight loss in the case of overweight or obesity, smoking cessation, reduced alcohol consumption and regular physical activity) and appropriate antihypertensive therapy is essential in order to avoid the maintenance of pathophysiological mechanisms favouring the persistence of low-grade inflammation in this particular subpopulation [71,72]. Thus, in this particular subtype of hypertensive patients at high cardiovascular risk highlighted in our study, the implementation of effective combined treatments for hypertension and moderate to severe OSAS with high intermittent hypoxia (adequate treatment with CPAP [≥4 h per night and ≥3 months] combined with lifestyle changes and appropriate management of hypertension) seems to be fundamental in order to enable better cardiovascular outcomes through a reduction in low-grade inflammation induced by this pathology [73].

### Limitations

Since the design of our study was retrospective, the results obtained must be confirmed by carrying out future prospective studies. In addition, since this study focused only on hypertension, its results cannot be generalised to other cardiovascular pathologies. Furthermore, despite the existence of other potential markers of low-grade inflammation, we decided to use CRP because this molecule is one of the most frequently used for patients with cardiovascular diseases and presents validated cut-offs to easily assess its potential impact on cardiovascular risk [31,74]. Finally, in order to avoid as much as possible any risk of selection bias during retrospective recruitment for this study, all hypertensive patients eligible according to the inclusion and exclusion criteria were included. Nevertheless, despite this systematic inclusion, only hypertensive patients who stayed consecutively at the Erasme Hospital Sleep Laboratory between 1 January 2002 and 31 December 2020 are present in our database, which may potentially limit the generalization of our results to all hypertensive patients.

## 5. Conclusions

In this study, we highlighted that low-grade inflammation is a frequent problem (33.8%) in hypertensive patients. Moreover, we demonstrated the existence of a particular subtype of hypertensive patients at high cardiovascular risk characterised by the presence of low-grade inflammation (CRP levels ≥ 3 mg/L but <10 mg/L) induced by high intermittent hypoxia related to moderate to severe OSAS. These results seem to indicate that adequate management (diagnosis and treatment) of moderate to severe OSAS with high intermittent hypoxia is essential in hypertensive patients to allow better cardiovascular outcomes. Finally, future prospective studies must be conducted to confirm the results of our study and to allow the development of new therapeutic strategies more specific to this particular subgroup of hypertensive patients.

## Figures and Tables

**Table 1:** Inclusion and exclusion criteria.

Inclusion Criteria	Exclusion Criteria
Hypertension according to the diagnostic criteria of the World Health Organization: mean systolic blood pressure ≥140 mmHg or mean diastolic blood pressure ≥90 mmHg or self-reported diagnosis of clinically demonstrated hypertension or taking antihypertensive medication	Acute and/or uncontrolled hepatic, pancreatic, pulmonary, cardiovascular, renal or infectious diseases
Age ≥ 18 years old	Past or current severe psychiatric diseases (psychotic disorder, bipolar disorder and substance use disorder)
CRP levels < 10 mg/L	Past or current cancer, autoimmune or inflammatory diseases
Absence of pregnancy	Past or current malformation or lesion (head trauma, lesions of cerebral respiratory centres, craniofacial malformations and abnormal chest deformities)
	Central disorders of hypersomnolence, OSAS already known or course of treatment before Sleep Laboratory and sleep apnoea syndrome with predominantly central component

CRP = C-reactive protein, OSAS = obstructive sleep apnoea syndrome.

**Table 2:** Summary of main results.

Analyses	Results
Comparison of polysomnographic parameters	Hypertensive patients with low-grade inflammation have higher parameters related to intermittent hypoxia (oxygen desaturation index and total time under 90% of oxygen saturation) than those without low-grade inflammation.
Prevalence of low-grade inflammation in hypertensive individuals	33.8%
Univariate analyses	Confounding factors identified: gender, body mass index, smoking, type 2 diabetes, dyslipidaemia status, aspirin therapy and Epworth Sleepiness Scale scores.
Multivariate analyses	Unlike mild OSAS and moderate to severe OSAS with low intermittent hypoxia, only moderate to severe OSAS with high intermittent hypoxia was significantly associated with higher risk of low-grade inflammation in hypertensive patients after adjustment for confounding factors.

OSAS = obstructive sleep apnoea syndrome.

## Data Availability

The data presented in this study are available on reasonable request from the corresponding author.

## Supplementary Materials

The following supporting information can be downloaded at: https://www.mdpi.com/article/10.3390/life14050592/s1, Annex S1: Procedure for outpatient recruitment of hypertensive patients; Annex S2: Blood pressure measurement method; Annex S3: Description of self-questionnaires; Annex S4: Stay conditions at the Sleep Laboratory and applied polysomnography montage; Annex S5: Scoring criteria for periodic limb movements; Annex S6: Description of the confounding factors included in the univariate analyses.

## Abbreviations

AHI	apnoea–hypopnoea index
CPAP	continuous positive airway pressure
CRP	C-reactive protein
DSM	Diagnostic and Statistical Manual of Mental Disorders
ODI	oxygen desaturation index
OSAS	obstructive sleep apnoea syndrome
