# Supplementary material for: Impact of Intermittent Hypoxia Related to Obstructive Sleep Apnoea Syndrome on Low-Grade Inflammation in Hypertensive Patients: Potential Implications for Cardiovascular Risk
Source: Life (Basel). 2024 May 6;14(5):592. doi: 10.3390/life14050592 (PMC11122566; doi:10.3390/life14050592)
Supplement: Supplementary file 1 [file life-14-00592-s001.zip › life-2975406-supplementary.pdf]

Supplementary Data

Annex 1

Procedure for outpatient recruitment of hypertensive patients

All hypertensive patients recruited for this study were initially referred to the Sleep Laboratory by physicians specialised in sleep medicine after an outpatient consultation during which a preliminary assessment of their complaints related to sleep, their ongoing psychotropic/somatic treatments and their somatic/psychiatric comorbidities was systematically performed to allow a first diagnostic hypothesis. Following this initial assessment, a polysomnographic recording was programmed in all these hypertensive patients to allow an objective assessment of their sleep complaints and to exclude the presence of comorbid sleep disorders that could negatively impact blood pressure control.

Annex 2

Blood pressure measurement method

Systolic and diastolic blood pressures were manually measured at the right arm after five minutes of rest in a sitting position by well-trained nurses. For subjects with a systolic blood pressure ≥140 mmHg and/or a diastolic blood pressure ≥90 mmHg, blood pressures were again measured twice after a systematic rest period of five additional minutes. The first measurement was excluded whereas the second and third measurements were averaged to minimise the impact of white coat effect. In the absence of prior diagnosis of hypertension, pathological blood pressures were confirmed by repeated measurements during the stay at the sleep laboratory.

Furthermore, after these repeated measurements of blood pressure in all subjects on antihypertensive medication, the presence of mean systolic blood pressure <140 mmHg and mean diastolic blood pressure <90 mmHg was used to define controlled hypertension whereas the presence of mean systolic blood pressure ≥140 mmHg and/or mean diastolic blood pressure ≥90 mmHg was used to define uncontrolled hypertension.

Annex 3

Description of the self-questionnaires

- The Beck Depression Inventory (reduced to 13 items) was used to investigate the presence of depressive symptoms. The 13 items of this scale may be scored from 0 to 3, which means that the total score may vary from 0 to 39. A final score of 0-4 indicates an absence of depressive symptoms, 5-7 mild depressive symptoms, 8-15 moderate depressive symptoms, and ≥16 severe depressive symptoms.

- The Epworth Sleepiness Scale was used to investigate daytime sleepiness. The 8 items of this scale assessing sleepiness in different daytime situations may be scored from 0 to 3, which means that the total score may vary from 0 to 24. A final score greater than 10 indicates excessive daytime sleepiness.

- The Insomnia Severity Index was used to investigate the severity of insomnia complaints. The 7 items of this index may be scored from 0 to 4, which means that the total score may vary from 0 to 28. A final score of 0-7 indicates an absence of insomnia complaints, 8-14 subclinical insomnia complaints, 15-21 moderate insomnia complaints, and 22-28 severe insomnia complaints.

Annex 4

Stay conditions at the Sleep Laboratory

The patients went to bed between 22:00 - 24:00 and got up between 6:00 - 8:00, following their usual schedule. During bedtime hours, the subjects were recumbent and the lights were turned off. Daytime naps were not permitted.

Applied polysomnography-montage

- Two electro-oculogram channels
- Three electroencephalogram channels
- One submental electromyogram channel
- Electrocardiogram
- Pressure cannula to detect the oro-nasal airflow
- Finger pulse-oximetry
- Microphone to record breathing sounds and snoring
- Plethysmographic inductive belts to measure thoracic and abdominal breathing
- Anterior tibialis electrodes

Annex 5

Scoring criteria for periodic limb movements

Periodic limb movements were scored based on the following strict criteria: 1) duration between 0.5 to 10 seconds, 2) interval between 5 and 90 seconds from leg movement onset and 3) movements had to be part of a series of ≥4 consecutive movements meeting these criteria. Periodic limb movement index corresponds to the total number of periodic limb movements divided by period of sleep in hours.

Annex 6

Description of the confounding factors included in the univariate analyses

After a review of the literature on factors associated with low-grade inflammation [1-10], potential confounders included in this study were body mass index (categorised: <30 kg/m², ≥30 kg/m²), age (categorised: <50 years, older ≥50 years), dyslipidaemia (categorised: no, untreated, treated), hypertension status (categorised: untreated, controlled, uncontrolled), number of antihypertensive treatment (categorised: 0, 1, 2, ≥3) insomnia disorders (categorised: no, sleep deprivation alone, insomnia without short sleep duration, insomnia with short sleep duration), sleep movement disorders (categorised: no, moderate to severe periodic limb movements during sleep, restless legs syndrome alone or combined with moderate to severe periodic limb movements during sleep), ESS scores (categorised: <11, ≥11 & <14, ≥14), major depression (categorical: no, remitted, current) and as binary variables: gender, smoking, alcohol, caffeine, type 2 diabetes, cardiovascular comorbidities and aspirin therapy.

References

1/Paiva, C.; Beserra, B.; Reis, C.; Dorea, J.G.; Da Costa, T.; Amato, A.A. Consumption of coffee or caffeine and serum concentration of inflammatory markers: A systematic review. *Crit Rev Food Sci Nutr*. **2019**, *59(4)*, 652-663.

2/Javaheri, S.; Redline, S. Insomnia and Risk of Cardiovascular Disease. *Chest*. **2017**, *152(2)*, 435-444.

3/Haapakoski, R.; Mathieu, J.; Ebmeier, K.P.; Alenius, H.; Kivimäki, M. Cumulative meta-analysis of interleukins 6 and 1β, tumour necrosis factor α and C-reactive

protein in patients with major depressive disorder. *Brain Behav Immun*. **2015**, *49*, 206-215.

4/Emerging Risk Factors Collaboration; Kaptoge, S.; Di Angelantonio, E.; Lowe, G.; Pepys, M.B.; Thompson, S.G.; Collins, R.; Danesh, J. C-reactive protein concentration and risk of coronary heart disease, stroke, and mortality: an individual participant meta-analysis. *Lancet*. **2010**, *375(9709)*, 132-140.

5/Osimo, E.F.; Cardinal, R.N.; Jones, P.B.; Khandaker, G.M. Prevalence and correlates of low-grade systemic inflammation in adult psychiatric inpatients: An electronic health record-based study. *Psychoneuroendocrinology*. **2018**, *91*, 226-234.

6/Andaku, D.K.; D'Almeida, V.; Carneiro, G.; Hix, S.; Tufik, S.; Togeiro, S.M. Sleepiness, inflammation and oxidative stress markers in middle-aged males with obstructive sleep apnea without metabolic syndrome: a cross-sectional study. *Respir Res*. **2015**, *16*, 3.

7/Cortez, A.F.; Muxfeldt, E.S.; Cardoso, C.R.; Salles, G.F. Prognostic Value of C-Reactive Protein in Resistant Hypertension. *Am J Hypertens*. **2016**, *29(8)*, 992-1000.

8/Jahn, C.; Gouveris, H.; Matthias, C. Systemic inflammation in patients with compromised upper airway anatomy and primary snoring or mild obstructive sleep apnea. *Eur Arch Otorhinolaryngol*. **2016**, *273(10)*, 3429-3433.

9/Gottlieb, D.J.; Somers, V.K.; Punjabi, N.M.; Winkelman, J.W. Restless legs syndrome and cardiovascular disease: a research roadmap. *Sleep Med*. **2017**, *31*, 10-17.

10/Kronish, I.M.; Rieckmann, N.; Shimbo, D.; Burg, M.; Davidson, K.W. Aspirin Adherence, Aspirin Dosage, and C-Reactive Protein in the First Three Months after an Acute Coronary Syndrome. *Am J Cardiol*. **2010**, *106(8)*, 1090-1094.
